# Comment on: “Atom‐Modified gDNA Enhances Cleavage Activity of TtAgo Enabling Ultrasensitive Nucleic Acid Testing”

**DOI:** 10.1002/advs.202406872

**Published:** 2025-03-08

**Authors:** Yue Tang, Xiao‐han Wang, Xu Xu, Lu‐sheng Xin, Xiu‐dan Wang, Xin‐min Li

**Affiliations:** ^1^ Institute of chronic diseases，Institute for Translational Medicine The Affiliated Hospital of Qingdao University School of Basic Medicine，College of Medicine Qingdao University Qingdao 266073 China; ^2^ State Key Laboratory of Marine Food Processing & Safety Control College of Food Science and Engineering Ocean University of China Qingdao 266404 China; ^3^ Jining Medical University Jining 272067 China

**Keywords:** 2'‐fluorine modified gDNA, dsDNA cleavage, gene editing, nucleic acid detection, TtAgo

## Abstract

The recent article by Zhang et al. piqued the interest. An atom‐modification‐based strategy is reported to enhance the cleavage activity of TtAgo, improving its practicability in TtAgo‐based nucleic acid testing. Specifically, the 2′‐fluorine (2′F)‐modified guide DNA (2′F‐gDNA) shows significant enhancement in the cleavage activity of TtAgo on double‐stranded (dsDNA) by increasing the melting temperature (Tm) and strengthening the binding affinity between 2′F‐gDNA and the targeted dsDNA. These findings are considered important for both molecular diagnostics and gene editing. A careful review of the article, however, raises questions that merit further discussion. After comprehensively reviewing the cleavage mechanism and structure of TtAgo‐gDNA‐target ternary complexes, and thoroughly analyzing our results, it is believed that the increased Tm and binding affinity of 2′F‐gDNA are not the primary factors that enhance cleavage activity, it is speculated that the 2′F modification at gDNA 3′‐end likely influences the propagation step. The data suggest that several details need to be addressed to improve the robustness of 2′F‐gDNA/TtAgo cleavage.

A recent article by Zhang et al.^[^ ^1^ ^]^ piqued our interest. They reported an atom‐modification‐based strategy to enhance the cleavage activity of TtAgo, improving its practicability in TtAgo‐based nucleic acid testing. Specifically, the 2′‐fluorine (2′F)‐modified guide DNA (2′F‐gDNA) was shown to significantly enhance the cleavage activity of TtAgo on double‐stranded (dsDNA) by increasing the melting temperature (Tm) and strengthening the binding affinity between 2′F‐gDNA and the targeted dsDNA. We believe these findings are important for both molecular diagnostics and gene editing.

TtAgo is guided by DNA (gDNA) to recognize and/or cleave targeted DNA or RNA. Compared to the CRISPR‐Cas system, TtAgo is more flexible and offers several advantages: i) it does not require specific protospacer adjacent motif (PAM) sequences in the targeted region, hence expanding the manipulable region of the DNA^[^ ^2^ ^]^; ii) the gDNAs are ≈16–21 nt in length, making them cheaper and more stable than RNA guides used in CRISPR‐Cas system;^[^ ^3^ ^]^ and iii) TtAgo is smaller in size and more compact. Therefore, the gDNA/TtAgo system holds significant potential for molecular diagnostics and gene editing.^[^ ^4^, ^5^ ^]^ Increasing numbers of TtAgo‐based nucleic acid detection strategies have been developed.^[^ ^6^, ^7^, ^8^, ^9^ ^]^ TtAgo has efficient cleavage activity for single‐stranded DNA (ssDNA); however, its poor cleavage activity on dsDNA and high reaction temperature requirement have hampered its applications.

In this recently published article, the authors aimed to enhance the cleavage activity of TtAgo on dsDNA using chemically modified gDNA.^[^ ^1^ ^]^ They proposed an atom‐modification‐based strategy, suggesting that the weak intermolecular forces among gDNA, TtAgo, and target DNA facilitate ternary complex formation. The substitution of 2′‐hydrogen in gDNA with a fluorine atom was proposed to increase the Tm and binding affinity of gDNA. In their experiments, the authors found that the cleavage activity of dsDNA increased significantly when the 2′F‐nucleotide modifications were placed at the 3′‐end of gDNA, reaching its maximum when gDNA had three modifications (3F‐gDNA). The authors claimed that the increased Tm and binding affinity of 2′F‐gDNA contributed to the enhanced cleavage activity.

A careful review of the article, however, raises questions that merit further discussion. First, the authors reported that 2′F‐gDNA enhanced the cleavage activity of TtAgo on dsDNA and lowered the reaction temperature, attributing these effects to improved Tm and binding affinity of the TtAgo‐gDNA complex. However, they did not explain the exclusive improvement in the cleavage activity of TtAgo with the 2′F modifications at the 3′‐end of the gDNA, while modifications at other positions or multiple sites showed no enhancement. The results indicated that increasing the number of modifications beyond the 3′‐end even inhibited its activity, which could not be explained by the Tm enhancement mechanism. We replicate the cleavage assay described by Zhang et al. (**Figure** **1**; Table S1, Supporting Information) (1). Our results showed that 5′‐end 2′F‐ modified gDNA (Figure 1, lanes 1 and 4) slightly inhibited the cleavage activity of TtAgo compared to that of canonical gDNA (Figure 1, lanes 2 and 5). Conversely, 3′‐end 2′F‐ modified gDNA slightly enhanced cleavage activity compared with the canonical gDNA (Figure 1, lanes 3 and 6). Only the 3′‐end modified 3F‐gDNA enhanced cleavage activity, despite both the 5′‐and 3′‐end modified 3F‐gDNA exhibiting similar increases in Tm value. Figure S4 (Supporting Information) in the published article by Zhang et al. showed that the MGB modification resulted in the highest Tm value among the various modifications. Given the authors’ claimed Tm values are important for cleavage activity, the MGB‐modified gDNA should have exhibited the highest cleavage activity. However, their result in **Figure** **2**D shows that TtAgo did not cleave dsDNA with the MGB‐modified gDNA,^[^ ^1^ ^]^ which contradicts their claim.

**Figure 1:**
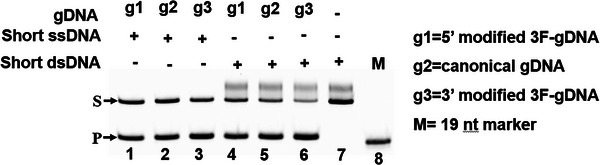
Comparison of the cleavage activity of TtAgo on short DNA with 2′F‐modified gDNAs. The “S” indicated the substrate, and “P” indicated the products.

**Figure 2:**
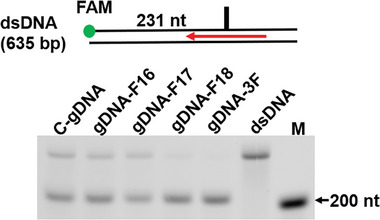
TtAgo cleavage assay with different gDNAs on long DNA in buffer with dNTPs. 2% alkaline agarose gel detecting the cleavage products of long dsDNA, the cleavage products were detected by the FAM signal on a cleaved strand.

Second, the dsDNA target used in the cleavage assay was only 30 bp. Given the reaction conditions in the article, it is possible that a large amount of dsDNA melted into ssDNA close to the Tm temperature.^[^ ^10^ ^]^To verify this, we incubated the 30 bp dsDNA at various temperatures and observed an increase in melted ssDNA as the incubation temperature rose from room temperature to 80 °C (Figure S1A, Supporting Information). Thus, concluding that 2′F‐gDNA enhances the cleavage activity of TtAgo on dsDNA could be questioned, and the possibility for short dsDNA melting into ssDNA during the cleavage assay must be eliminated. We addressed this interference by preparing 635 bp dsDNA using a polymerase chain reaction (Table S1, Supporting Information) and confirmed that this longer dsDNA did not melt into ssDNA under TtAgo cleavage conditions (Figure S1B, Supporting Information). Next, we optimized the TtAgo cleavage conditions using a molecular beacon with a FAM and TAMRA FRET pair (Figure S2A, Supporting Information).^[^ ^11^ ^]^ Our results indicated that TtAgo assembled more effectively with gDNA at room temperature (Figure S2B (lines 3–6),C, Supporting Information), and the dNTP significantly enhanced the TtAgo cleavage activity(Figure S2B (lines 1–4),C, Supporting Information), which was consistent with the findings of Song et al.^[^ ^12^ ^]^ We prepared canonical gDNA of lengths 16,18, and 20 nt (gDNA‐C16, gDNA‐C18, and gDNA‐C20), 2′F‐modified 18 nt gDNAs at the 16th, 17th, and 18th positions (gDNA‐F16, gDNA‐F17, and gDNA‐F18), and a modified gDNA with three 2′F‐nucleotides at the 3′‐end (gDNA‐3F) (Table S1, Supporting Information). The TtAgo cleavage activity on the 635 bp dsDNA with different gDNAs was tested under the optimized conditions (Figure 2). Our results showed that only the 2′F modifications at the 3′‐end of gDNA enhanced the TtAgo cleavage activity (Figure 2), even though gDNA‐F16 and gDNA‐F17 should have similarly increased the Tm value as gDNA‐F18. The 2′F‐modified gDNA did not significantly enhance cleavage when dNTPs were removed from the cleavage buffer (Figure S3, Supporting Information).

We also assessed the cleavage activity of TtAgo using gDNA‐C16, gDNA‐C18, and gDNA‐C20 (Table S1, Supporting Information). The results revealed that cleavage activity did not increase, despite an expected rise in Tm with longer gDNA (Figure S4, Supporting Information). Similar findings were reported by Song et al.^[^ ^12^ ^]^ These results indicate that the increased Tm of gDNA is unlikely the primary reason for enhancing TtAgo cleavage activity.

Third, the schematic of the FAST assay (Zhang et al., Figure 4) indicates that the FAST template could serve as the target for the secondary cleavage reaction. The 5′‐phosphorylated red and grey sequences produced in step (v) are complementary to the FAST template, and this newly generated 5′‐phosphorylated ssDNA is recognized by TtAgo as a new gDNA.^[^ ^8^, ^13^ ^]^ Therefore, TtAgo may cleave the FAST template at the red and gray sequence regions, separating the G‐quadruplex generation template from the miRNA binding site. However, Zhang's data showed that these secondary cleavages do not significantly interfere with the FAST amplification process, one possible explanation is that the limited concentration of TtAgo and the stronger activity of F‐gDNA/TtAgo hindered the occurrence of the secondary cleavages of the FAST template, which should be analyzed by the authors.

After comprehensively reviewing the cleavage mechanism and structure of TtAgo‐gDNA‐target ternary complexes,^[^ ^3^, ^14^, ^15^, ^16^ ^]^ and thoroughly analyzing our results (Figures 1 and 2; Figures S3 and S4, Supporting Information), we believe that the increased Tm and binding affinity of 2′F‐gDNA are not the primary factors that enhance cleavage activity. During TtAgo cleavage, the 3′ end of the gDNA is well‐known to be released from the PAZ pocket, which aids the transition from a cleavage‐incompatible to a cleavage‐compatible state. In the propagation step, the release of the gDNA 3′‐end from the PAZ domain is accompanied by a conformational change in E512, forcing E512 to complete the Asp‐Glu‐Asp‐Asp catalytic tetrad formation.^[^ ^16^ ^]^ Therefore, we speculate that the 2′F modification at gDNA 3′‐end likely influences the propagation step. This hypothesis could be further validated by analyzing the structure of TtAgo‐gDNA‐target ternary complexes.

We commend Zhang et al. for drawing attention to novel approaches to improve the cleavage activity of TtAgo, which would be an important advancement not only in molecular diagnostics but also in gene editing and therapeutics. Achieving improved gDNA modification that enhances cleavage activity at lower reaction temperatures could enable TtAgo to effectively cleave dsDNA at lower or even physiological temperatures, making TtAgo a promising tool for convenient molecular diagnostic and gene therapy applications. However, the mechanism of 2′F‐gDNA enhancing the TtAgo cleavage activity should be discussed more thoroughly, and our data suggest that several details need to be addressed to improve the robustness of 2′F‐gDNA/TtAgo cleavage, such as gDNA sequence, length, and reaction buffer conditions. The degradation of the 2′F modification may also influence the robustness of 2′F‐gDNA/TtAgo cleavage, a potential solution could be the use of nuclease‐resistant modified 2′‐fluoro‐phosphorothioate gDNA.^[^ ^17^ ^]^

## Conflict of Interest

The authors declare no conflict of interest.

## Author Contributions

Y.T., X.W., and X.X. contributed equally to this work.

## Supporting information



Supporting Information
