# Supplementary material for: Comment on: “Atom‐Modified gDNA Enhances Cleavage Activity of TtAgo Enabling Ultrasensitive Nucleic Acid Testing”
Source: Adv Sci (Weinh). 2025 Mar 8;12(13):2406872. doi: 10.1002/advs.202406872 (PMC11967831; doi:10.1002/advs.202406872)
Supplement: Supplementary file 1 — Supporting Information [file ADVS-12-2406872-s001.docx]

**Supporting Information**

Comment on: “Atom-Modified gDNA Enhances Cleavage Activity of TtAgo Enabling Ultra-Sensitive Nucleic Acid Testing”

Yue Tang^1^, Xiao-han Wang^1^, Xu Xu^2^, Lu-sheng Xin^3^, Xiu-dan Wang^2^*, Xin-min Li^1^*

1, Institute of chronic diseases, Institute for Translational Medicine, The Affiliated Hospital of Qingdao University, School of Basic Medicine, College of Medicine, Qingdao University, Qingdao, 266073

2, State Key Laboratory of Marine Food Processing & Safety Control, College of Food Science and Engineering, Ocean University of China, Qingdao, 266404

3, Jining Medical University, Jining, 272067

**⁎**Corresponding Authors,

E-mail addresses: wangxiudan@ouc.edu.cn (Xiu-dan Wang), [xinminli@qdu.edu.cn](mailto:xinminli@qdu.edu.cn) (Xin-min Li)

**Materials and Methods**

***Materials*** The ssDNA target, gDNAs, Molecular Beacon (MB) and PCR primers were purchased from Shanghai Sangon Biotech (Shanghai, China), and acrylamide/methylene acrylamide (19:1), 10× TBE buffer, urea and PCR kit were also purchased from Shanghai Sangon Biotech (Shanghai, China), GeneGreen Nucleic Acid Dye and DNA Clean Up kits were purchased from TIANGEN Biotech (Beijing)Co.,Ltd.(# DP204-02), TtAgo was purchased from NEB (#M0665S). TtAgo in pet28a was synthesized by Tsingke Biotech Co., Ltd. (Beijing, China).

***Preparation of dsDNA target*** ***and purification of TtAgo*** dsDNA target was prepared with Primer-F and Primer-R as described in PCR kit, and purified with DNA Clean Up kits. TtAgo was purified as previous described (1).

***Optimizing the reaction of TtAgo cleavage*** The TtAgo (0.2 μM) and gDNAs (1 μM) were assembled in 1x thermoPol reaction buffer (20 mM Tris-HCl pH 8.8,10 mM (NH_4_)_2_SO_4_, 10 mM KCl, 2 mM MgSO_4_, 0.1% Triton® X-100), and the dNTPs was added as illustrated in figure S2, followed by the addition of MB. The mixture was incubated for another 150 minutes, and the FAM signal was recorded by QuantStudio 3 Real-Time PCR System. The cleavage products were quenched by qual 2×TBE-urea buffer, and the products were resolved on a denaturing 20% PAGE gel and stained with gel red. The gel was visualized the signal using a Bio-rad universal hood II Imager.

***TtAgo Cleavage Assay*** Cleavage assay was carried out according to the instructions. Briefly, the TtAgo (0.1 μM) and gDNAs (0.5 μM) were assembled in 1x thermoPol reaction buffer (20 mM Tris-HCl pH 8.8,10 mM (NH_4_)_2_SO_4_, 10 mM KCl, 2 mM MgSO_4_, 0.1% Triton® X-100) at room temperature for 30 minutes, and 0.2 mM dNTP was added as illustrated in figures, followed by addition of 0.1 μM short ssDNA/short dsDNA or 20 ng long dsDNA. And the mixture was incubated for another 30 minutes. The short ssDNA/short dsDNA cleavage reaction was quenched by equal volume stop buffer (95% deionized formamide, 40 mM NaOH), and the products were resolved on a denaturing 20% PAGE gel and the labeled FAM signal was visualized through a Typhoon RGB Imager (GE Healthcare). the long dsDNA cleavage reaction was quenched by alkaline gel-loading buffer (300 mM NaOH, 6mM EDTA, 18% (w/v) Ficoll 400), and the products were resolved on a 2% alkaline agarose gel and visualized by FAM through a Typhoon RGB Imager (GE Healthcare)(2).

Table S1. The DNA sequences used in TtAgo cleavage assay

| Name | Sequences(5’to3’) |
| --- | --- |
| 5′FAM-Template DNA (short ssDNA) | CTGCAGTCGTCGTAGCTGATCGATGCATGC |
| Complementary DNA | 5'-GCATGCATCGATCAGCTACGACGACTGCAG |
| 5′-end modified 3F-gDNA | 5'p-C/i2FA//i2FU//i2FG/CATCGATCAGCTAC |
| canonical gDNA | 5'p-CATGCATCGATCAGCTAC |
| 3′end modified 3F-gDNA | 5'p-CATGCATCGATCAGC/i2FU//i2FA//i2FC/ |
| Molecular Beacon (MB) | FAM-cgcaccAAATGATACGCAGCGAggtgcg-TAMRA |
| MBg-DNA | 5’P- TCGCTGCGTATCATTT |
| Long dsDNA | FAM- GGCTTCGGAGTCCCCTGCCATTACGATACACGTCTGCAGTCAACTGGAATTTTCATGATTGAATTTTGTAAGGTATTTTGAAATAATTTTTCATATAAAGGTGAGTTTGTATTAAAAGGTACTGGTGGAGTATTTGATAGTGTATTAACCTTATGTGTGACATGTTCTAATATAGTCACATTTTCATTATTTTTATTATAAGGCCTGCTGAAAATGACTGAATATAAACTTGTGGTAGTTGGAGCTGGTGGCGTAGGCAAGAGTGCCTTGACGATACAGCTAATTCAGAATCATTTTGTGGACGAATATGATCCAACAATAGAGGTAAATCTTGTTTTAATATGCATATTACTGGTGCAGGACCATTCTTTGATACAGATAAAGGTTTCTCTGACCATTTTCATGAGTACTTATTACAAGATAATTATGCTGAAAGTTAAGTTATCTGAAATGTACCTTGGGTTTCAAGTTATATGTAACCATTAATATGGGAACTTTACTTTCCTTGGGAGTATGTCAGGGTCCATGATGTTCACTCTCTGTGCATTTTGATTGGAAGTGTATTTCAGAGTTTCGTGAGAGGGTAGAAATTTGTATCCTATCTGGACCTAAAAGACACACTCGCCGCAGGCTGG |
| Primer-F | FAM-GGCTTCGGAGTCCCCTGC |
| Primer-R | CCAGCCTGCGGCGAGTG |
| gDNA -C16 | p-TGCCTACGCCACCAGC |
| gDNA -C20 | p-TGCCTACGCCACCAGCTCCA |
| gDNA -C18(C-gDNA) | p-TGCCTACGCCACCAGCTC |
| gDNA -F16 | p-TGCCTACGCCACCAG/i2FC/TC |
| gDNA -F17 | p-TGCCTACGCCACCAGC/i2FU/C |
| gDNA-F18 | p-TGCCTACGCCACCAGCT/i2FC/ |
| gDNA-3F | p-TGCCTACGCCACCAG/i2FC//i2FU//i2FC/ |

The gDNAs binding sites are highlighted with underlines, p represents 5′-phosphorylate modification, /i2FC/, /i2FU/ represents 2′F-modified C and U, respectively.


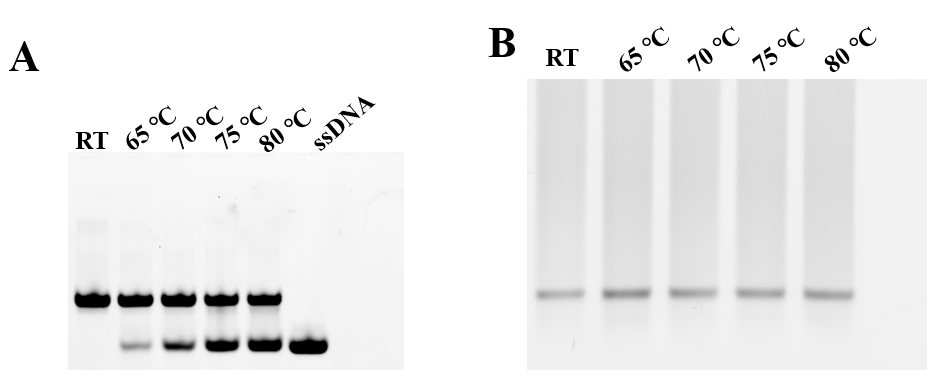


Figure S1. Detecting the stability of dsDNA in TtAgo cleavage conditions by Gel electrophoresis. A, 10% native PAGE gel detected the melted short ssDNA when improved the incubation temperature from room temperature to 80℃. B, the long dsDNA (635 bp) did not melt to ssDNA under TtAgo cleavage conditions.


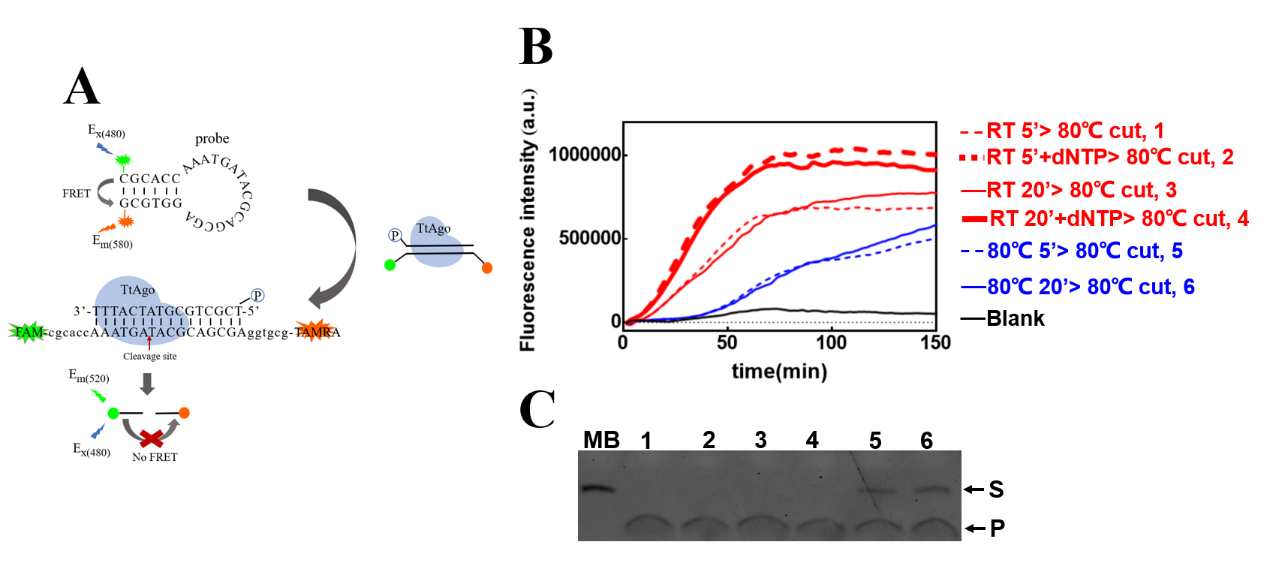


Figure S2. Optimizing the TtAgo cleavage conditions using molecular beacon (MB) based on FAM and TAMRA FRET pair. A, Schematic of the MB sensing the TtAgo cleavage. B, The curves indicating the optimized TtAgo cleavage conditions:1, TtAgo assembled with gDNA at room temperature 5 min, then cleaved at 80℃; 2, added dNTP in buffer, TtAgo assembled with gDNA at room temperature 5 min, then cleaved at 80℃; 3, TtAgo assembled with gDNA at room temperature 20 min, then cleaved at 80℃; 4, added dNTP in buffer, TtAgo assembled with gDNA at room temperature 20 min, then cleaved at 80℃; 5, TtAgo assembled with gDNA at 80℃ 5 min, then cleaved at 80℃; 6, TtAgo assembled with gDNA at 80℃ 20 min, then cleaved at 80℃; C, 20% denaturing gel detecting the cleavage products in B.


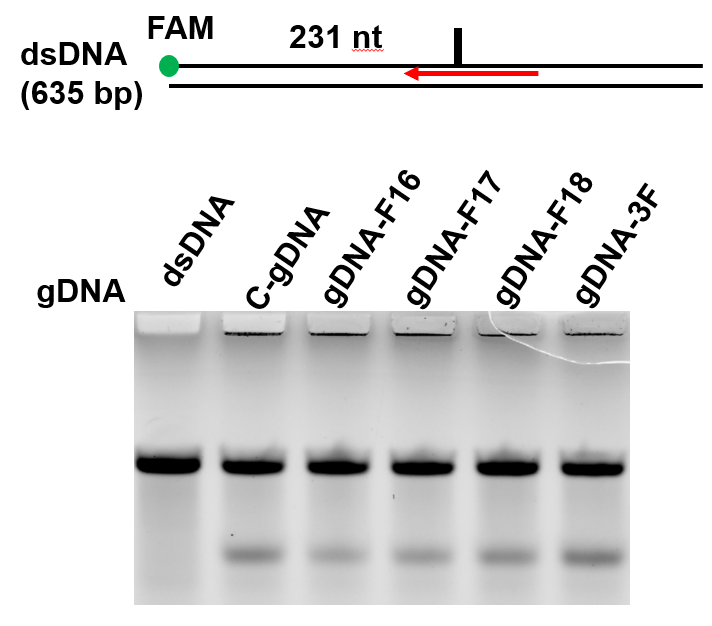


Figure S3. TtAgo cleavage assay with different gDNAs on long DNA in buffer without dNTPs. 2% alkaline agarose gel the cleavage of long dsDNA by different gDNAs, the cleavage products were detected through the FAM signal on cleaved strand.


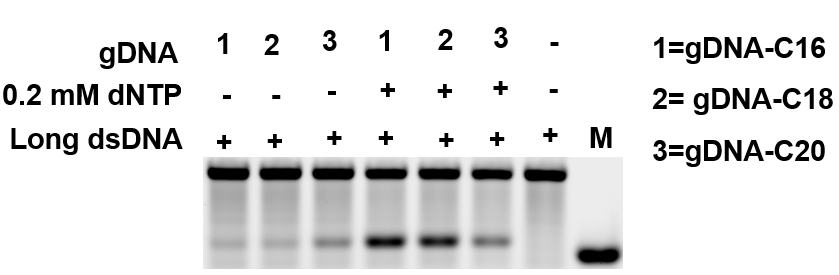


Figure S4. TtAgo cleavage assay with different length gDNAs on long DNA.

**References**

1. Fang, J., Yuan, C., Luo, X., He, Z. and Fu, W. (2024) A Thermus thermophilus argonaute-coupling exponential amplification assay for ultrarapid analysis of circulating tumor DNA. *Talanta*, **266**, 125034.

2. Green, M.R. and Sambrook, J. (2021) Alkaline Agarose Gel Electrophoresis. *Cold Spring Harbor protocols*, **2021**.
